# Recent Advances in Studying Interfacial Adsorption of Bioengineered Monoclonal Antibodies

**DOI:** 10.3390/molecules25092047

**Published:** 2020-04-28

**Authors:** Peter Hollowell, Zongyi Li, Xuzhi Hu, Sean Ruane, Cavan Kalonia, Christopher F. van der Walle, Jian R. Lu

**Affiliations:** 1 Biological Physics Laboratory, Department of Physics and Astronomy, University of Manchester, Oxford Road, Schuster Building, Manchester M13 9PL, UK; peter.hollowell@postgrad.manchester.ac.uk (P.H.); zongyi.li@manchester.ac.uk (Z.L.); xuzhi.hu-3@postgrad.manchester.ac.uk (X.H.); sean.ruane@postgrad.manchester.ac.uk (S.R.); 2 Dosage Form Design & Development, BioPharmaceutical Development, BioPharmaceuticals R&D, AstraZeneca, Gaithersburg, MD 20878, USA; cavan.kalonia@astrazeneca.com; 3 Dosage Form Design & Development, BioPharmaceutical Development, BioPharmaceuticals R&D, AstraZeneca, Cambridge CB21 6GH, UK; christopher.x.vanderwalle@gsk.com

**Keywords:** mAbs, surface adsorption, co-adsorption, antibody, structural unfolding, self-assembly, surfactant, neutron reflection

## Abstract

Monoclonal antibodies (mAbs) are an important class of biotherapeutics; as of 2020, dozens are commercialized medicines, over a hundred are in clinical trials, and many more are in preclinical developmental stages. Therapeutic mAbs are sequence modified from the wild type IgG isoforms to varying extents and can have different intrinsic structural stability. For chronic treatments in particular, high concentration (≥ 100 mg/mL) aqueous formulations are often preferred for at-home administration with a syringe-based device. MAbs, like any globular protein, are amphiphilic and readily adsorb to interfaces, potentially causing structural deformation and even unfolding. Desorption of structurally perturbed mAbs is often hypothesized to promote aggregation, potentially leading to the formation of subvisible particles and visible precipitates. Since mAbs are exposed to numerous interfaces during biomanufacturing, storage and administration, many studies have examined mAb adsorption to different interfaces under various mitigation strategies. This review examines recent published literature focusing on adsorption of bioengineered mAbs under well-defined solution and surface conditions. The focus of this review is on understanding adsorption features driven by distinct antibody domains and on recent advances in establishing model interfaces suitable for high resolution surface measurements. Our summary highlights the need to further understand the relationship between mAb interfacial adsorption and desorption, solution aggregation, and product instability during fill-finish, transport, storage and administration.

## 1. Why is Studying Monoclonal Antibody Adsorption Important?

Biopharmaceuticals are highly versatile and over the past ten years or so, bioengineered proteins, especially monoclonal antibodies (mAbs), have emerged as important drugs in treating several major disease areas, including immune checkpoint inhibitors (immuno-oncology) and treatments for chronic diseases [1]. The industrial process to produce therapeutic mAbs can remove aggregation and other impurities during the downstream process but problems can be encountered when there are incompatibilities with interfaces during fill-finish, transportation, storage and administration. Interactions with interfaces can result in denaturation of the antibody and potential sub-visible particle formation [2,3,4,5,6]. The design of mAbs rarely involves considerations for the effects of interfacial adsorption, to this end, products can sometimes face delays when incompatibilities between mAb and interfaces result in internal specification failures. Such failures can result in product batches being discarded, which is expensive and can delay important treatments to market. Studying mAb adsorption at interfaces where incompatibilities are detrimental to production could provide an insight in what is triggering unwanted aggregation, and this knowledge could be used to guide how therapeutics are formulated/designed to help prevent batch failures caused by material incompatibilities and related project delays.

The therapeutic protein market has been steadily gaining traction [7] and in 2017 the global therapeutic monoclonal antibody (mAb) market was worth approximately USD 108 billion with an expected generated revenue by the end of 2023 around USD 219 billion [8]. The majority of current mAb therapeutics are derived from natural IgG1 proteins, with sequence modifications in Fab or Fc or both. In spite of extensive efforts made in their development, there is no assurance about their stability under different stages of production and application. Lack of structural stability can cause a range of issues as mAbs at high concentrations (> 100 mg/mL) tend to aggregate, triggering the growth of larger aggregates and possible precipitates. Protein adsorption and desorption constitutes a crucial step that may induce structural instability and promote aggregate nucleation and growth mechanisms. Given the fast expansion in the development of protein drugs, a good way to reduce risk when developing products is by considering adsorption before challenges emerge in late stage development. Following the strict rules imposed by regulatory filing and review, a protein drug must remain stable over its stated lifetime, contain no more than 6000 sub-visible particles ≥ 10 µm, 600 sub-visible particles ≥ 25 µm per container, as well as harbour practically no visible particulates [9].

This review aims to inform the reader about how mAb adsorption occurs and how this process may be relevant to therapeutic mAb production based on recent studies. A general introduction to the topic will be provided, followed by a description of key spectroscopic and imaging techniques used to examine protein adsorption. The impact of different interfacial adsorption processes will be discussed in the context of industrial bioprocessing and exemplified by recent studies of mAb adsorption at air/water, solid/water and oil/water interfaces. With regard to high resolution interfacial measurement, the focus will be on neutron reflection, a technique that can provide accurate depth and composition measurements at Ångstrom level, thereby allowing inference about the physical states of the adsorbed mAb molecules. An outlook to future developments in interfacial science as applied to proteins will finally be provided.

## 2. Biopharmaceutical Production

This section will introduce the stages in producing biopharmaceuticals and highlight when interfacial adsorption can become problematic.

### 2.1. Upstream Process

Initially, in biopharmaceutical production, a stock culture is prepared with cells able to produce recombinant mAb. After optimizing parameters such as temperature, pH and the type of process, large scale bioreactors are used to acquire large quantities of the desired mAb. Separation of mAbs as target proteins from the rest of cell masses can be achieved by precipitation, centrifugation and porous membrane filtration. Most porous membranes used in different types of filtration are fabricated from polymeric materials, with pore sizes ranging from micro to nanometre range. Separation is largely based on effective pore sizes. Interactions between mAb and pore surface via entropic, hydrophobic and electrostatic effects influence how a given mAb adsorbs and how adsorbed mAb layer inside the pore surface mediates mAb permeation and subsequent blockage or filter fouling [10].

### 2.2. Downstream Process

At the present stage, mAb purification is often carried out by different forms of liquid chromatography wherein the clarified cell media is first passed through a packed porous resin derivatized by protein A to capture the Fc, followed by orthogonal purification processes utilizing different protein surface charge (mAbs typically have a pI > 7.5) to further remove host cell protein and a final polishing step [11,12,13,14]. Like porous membrane separation, these column based separations also expose the mAb to very high surface areas that are potentially destabilizing as a consequence of adsorption/desorption. Viral clearance is required to reduce the risk of known, or unknown, viral contaminants. There are several methods used to achieve this including low pH treatments, detergent inactivation viral filtration as well as viral clearance steps such as protein A chromatography [10,15].

Highly purified proteins are often unstable in solution because they have been removed from their native environments (e.g., serum or cytosol) which supported their native structures. In addition, many mAbs are engineered, containing large segments of artificially designed sequences (e.g., scFv) that may not form well folded structures. Formulation is a process that improves mAb stability by adding buffer/salt ions, amino acids, polyols and non-ionic surfactants [16]. The mAb drug substance will be filled into bulk plastic containers and may be frozen for long term storage.

### 2.3. Fill/Finish

The drug substance is shipped to the fill site and thawed if it was frozen. The drug substance is generally temperature equilibrated, pooled, mixed and sterile-filtered before being filled into glass vials, pre-filled syringes or related devices with a target shelf-life of greater than 18 months. Adsorption and desorption at solid/water and air/water interface can negatively impact product quality.

Shaking and shearing of a liquid product in transit are inevitable and will cause the mAb to be adsorbed/desorbed at the expanding/contracting air/liquid interface, respectively, also potentially leading to aggregation in solution [17]. Thus, apart from adsorption and desorption onto different substrate surfaces, the process at the air/water interface can also underline mAb stability.

**Figure 1:**
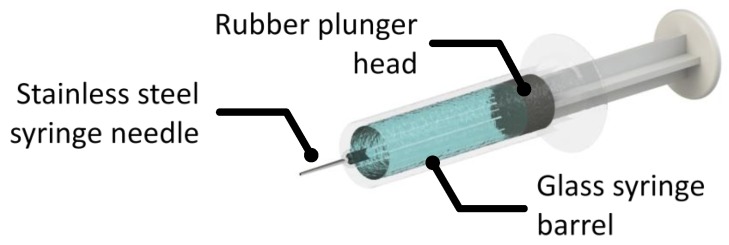
An illustration of a basic syringe system with a stainless steel needle, a glass barrel and lubricated rubber plunger.

Finally, it is useful to comment on primary containers. As parenteral administration is often necessary, tremendous effort has been put on improving administrative efficiency, reliability and convenience. The development of prefilled syringes helps overcome the lack of convenience, accuracy and some safety concerns that come with traditional parenteral administration. The interfaces present in prefilled syringes are presented in Figure 1. There can also be residual tungsten from the tip forming process. The syringe needle is generally stainless steel and many syringe barrels are made of glass or plastics (polyolefins). To ensure suitable break loose and glide forces, prefilled syringe plunger heads and the syringe barrel are often coated in silicone oil for lubrication. This approach has been incorporated into the formal surface preparation protocols for vial stoppers and syringes used for mAb storage and administration.

For products administered in the clinic, closed system transfer devices and IV bags can pose further challenges with regards to product-surface incompatibilities. Correct formulation of the final mAb product in aqueous solution is important to prevent-product adsorption and sub-visible or visible particle formation [18,19,20].

## 3. Introduction to Proteins, Monoclonal Antibodies and Adsorption

Proteins are polymers of amino acids that fold into well-defined local constructs such as helical strands, sheets and turns and three-dimensional (3D) structures. Structural folds are complex but proteins in their native states usually have well-defined 3D, or tertiary, structures with hydrophobic amino acids buried inside and polar and charged amino acids projected outside. Because of the anisotropic distribution of charged, polar, and apolar groups, some parts of a protein’s surface are more hydrophobic than other parts. These amphiphilic molecules spontaneously adsorb when exposed to interfaces. Fouling of surgical equipment, spoiling of contact lenses and contamination in food process plants [21,22,23,24,25,26,27] represent examples where protein adsorption imposes burden to technological processes. On the other hand, protein adsorption can be beneficial as well as hindering. For example, fabrication of functional proteins onto detection surfaces of sensors and surfaces of implants is crucial in ensuring product reliability. Various technical processes must be deployed to either promote protein adsorption where desired or inhibit protein adsorption where undesired, but in all cases, the instability of proteins, often induced by adsorption/desorption processes, causes complications and adds uncertainty in either removing fouled proteins in the former or sustaining in protein bioactivities in the latter. IgG antibodies are proteins that provide the body immunity agaist pathogens. They are comprised of 4 peptide chains and come in 4 sub classes with differences lying in their location and number of interchain-disulphide bonds and their main role in the body is to initiate phagocytosis. Initiated mechanisms include complement-dependant cytotoxicity (CDC), antibody-dependent cellular cytoxicity (ADCC) and receptor blocking. CDC can spontaneously identify pathogens, the classical pathway is initiated by the binding of the antibody Fc to C1q protein. This results in a membrane attack complex and target cell lysis [28]. ADCC is a cell mediated immune respose whereby a target cell is activly lysed. Antibodies bind to antigens on the target cell surface, then most commonly a natrual killer cell’s Fc receptor (CD16) will bind to the Fc region of the antibody resulting in lysing of the unwanted cell [29]. Blocking antibodies prevents other antibodies from binding to an antigen. One such mAb, Ipilimumab, works by reducing the down regulation of the immune sytsem, thereby activating the immune system [30].

### 3.1. Structure of Bioengineered IgG mAbs

Development of bioengineered mAbs has enabled a biotherapeutic market to emerge, here will be discussed the structure of an example IgG1 commonly studied. It is important to note that protein crystalline structure is significant to the study of protein behaviour and can be acquired by X-Ray Diffraction (XRD) [31], cryo-electron microscopy (cyro-EM) [32] and nuclear magnetic resonance (NMR) [33].

**Figure 2:**
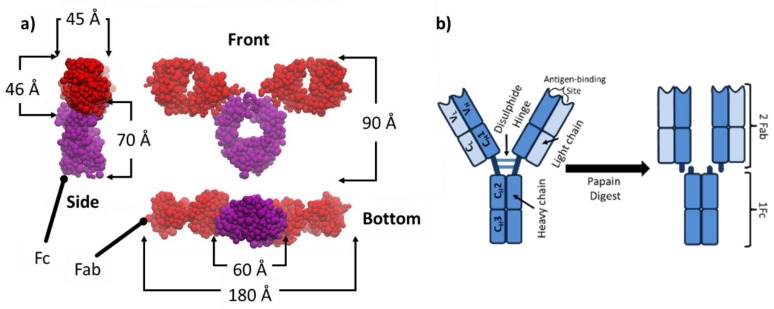
(**a**) Molecular model of COE-3 taking after the native human IgG1κ. Side view, front view and bottom view projected from the space filled IgG1κ model. (**b**) The schematic depiction of the molecule, its key domains and the cleavage of the hinge to produce Fab and Fc fragments via papain digestion.

A model bioengineered mAb that has been extensively studied is designated as COE-3. It is very similar to the humanized IgG1k 1HZH whose crystalline structure has been determined with high resolution [34] and the side view, front view and bottom view are shown in Figure 2a, with the approximate dimensions of 45 × 90 × 180 Å. The full-length human IgG1κ has the molecular weight of 145,560 Da (assuming average glycans) and an isoelectric point (pI) of 8.44. The molecule contains several regions: Two Fab (fragment antigen-binding) and one Fc (fragment crystallizable), linked together by a disulphide hinge, as schematically shown in Figure 2b. The Fc of COE-3 has the same sequence as IgG1κ but its Fab region has only ≈75% sequence identity to the wild type [35]. One Fc and two Fab fragments can be obtained from one COE-3 molecule through papain digestion (enzymatic cleavage) by cutting the hinge as illustrated in Figure 2b; this enables us to examine the adsorption behaviour of the individual domains. The free Fc and Fab segments can be separated and purified from the digestion product by chromatography (protein A affinity capture followed by ion exchange) and then buffer exchanged and concentrated by ultrafiltration. Bioengineered mAbs do not have to be confined to the same domain architecture and therefore their molecular weights deviate from wild type antibodies. Depending on design principles, mAbs may be engineered with additional single chain variable fragment (scFv) to impart bispecific functionality [36]. In the context of this review, we will focus on mAbs with molecular weights close to the wild type IgG1.

### 3.2. Main Techniques Used for Studying mAb Adsorption

The underpinning molecular events involved in mAb adsorption and desorption are complex and not yet fully predictable. A number of physical and analytical techniques have been developed to gain information about different interactions related to adsorption processes directly and indirectly [37]. Imaging techniques such as scanning electron microscopy (SEM) and atomic force microscopy (AFM) are used to study the patterns of adsorbed mAb molecules at the solid/liquid interface. A series of spectroscopic techniques, based on both optical and neutrons as well as X-rays, have been well developed to probe the adsorbed mAb layer, investigating the layer thickness, layer structure and the secondary structure of the adsorbed mAb within the layer. For the adsorption at the air-water interface, surface pressure measurements have also been used to monitor changes in surface tension with time, pressure and influence from any other additives in solution [17]. Studies have aimed to investigate fundamental driving forces of protein adsorption with many also trying to link experiments with molecular dynamics simulations [38,39,40].

In addition to the assessment of adsorption and desorption of proteins, there have also been studies into methods that reduce or control mAb adsorption, e.g., by adding excipients into solution or changing the chemistry or morphology of the substrate interface, with some to be covered in this review. This section aims to provide an overview of several key techniques that are used to study protein adsorption.

#### 3.2.1. Spectroscopic Techniques

Fourier transfer infrared spectroscopy (FTIR) is an important tool to investigate the secondary structures and secondary structural changes of proteins to help understand how its instability occurs. Most FTIR instruments use a Michelson interferometer setup to gather information about the absorption of infrared light at multiple wavelengths. A region in the infrared band known as the amide I region (1600–1700 cm^−1^) is governed by the stretching of C=O and C-N, i.e., the protein backbone conformation and hydrogen bond pattern. Hence, it contains the information of the secondary structure of a protein sample. The shape of the absorption in the amide I region can be deconvoluted to provide the fraction of the proteins in different secondary structures, including α-helices, β-sheets and random coils. Special auxiliary parts are fitted to various FTIR systems to obtain measurements at the air/water, air/solid and liquid/solid interfaces, making the IR information complementary to interfacial data obtained from other techniques. Observations over time can illustrate protein’s ‘relaxing’ at the interface from the changes in the secondary structure.

**Figure 3:**
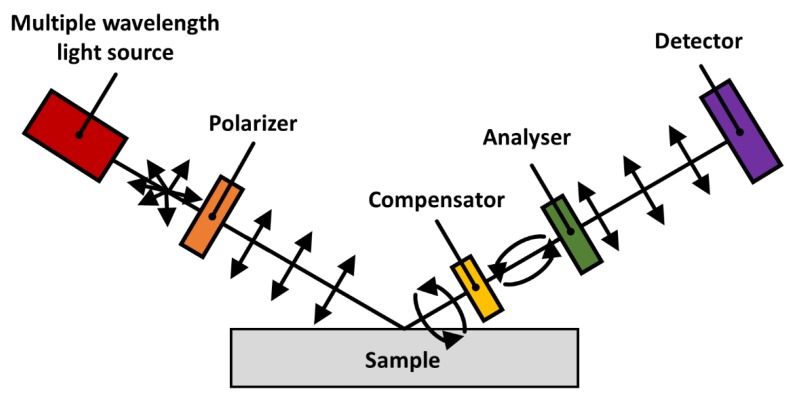
Simple schematic of J.A. Woollam 2000 M spectroscopic ellipsometer, including (from left to right) a QTH deuterium light source and a 75W xenon light source, followed by a fixed calcite Glan-Taylor polarizer. Next is a continuously rotating compensator then a fixed calcite Glan-Taylor polarizer analyser. Finally, a back-thinned silicon CCD array detector is used to measure UV and visible light and an InGaAs photodiode array detector is used to measure near infrared.

Spectroscopic ellipsometry (SE) exploits the polarized nature of light to measure the dielectric properties of thin films, which can in turn be used to measure the adsorbed mass of protein on reflective surfaces. A standard SE setup configuration is as follows: a multiple wavelength light source(s) is polarized and reflected off the sample at either single or multiple angles; an optional compensator can enhance measurement accuracy; after passing through a birefringent material to polarize the light it is finally detected. When light, of a known polarized nature, is reflected from a surface with a deposited sample film, changes in amplitude and phase measured by a CCD array and near infrared detector can be used to investigate the nature of the film over a range of wavelengths. For a schematic diagram of a SE, see Figure 3.

**Figure 4:**
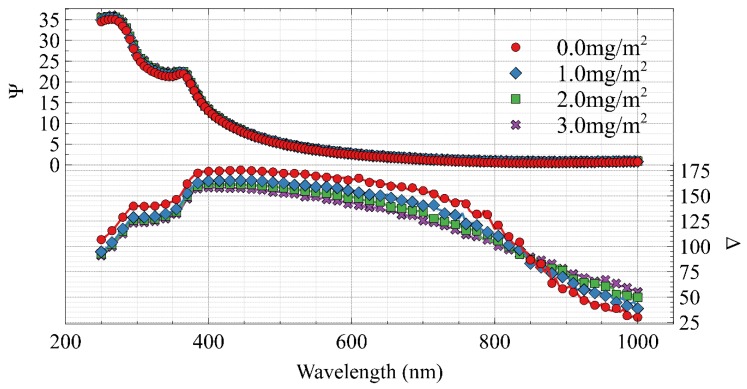
Simulated data showing increasing adsorbed amounts at the SiO_2_/water interface. There is sensitivity to the adsorbed amount in the phase information, Δ, as well as a subtle but distinct shift in the Ψ data set. The simulation used a silicon substrate with a native oxide layer of 13Å and a Cauchy protein model in H_2_O.

Using known constants and dispersion relationships and probing the surface or interface with multiple wavelengths of electromagnetic radiation (spectroscopic ellipsometry) the adsorbed amount of a protein can be determined at a given interface. Common parameters used to study non-birefringent systems are Ψ and Δ. These are defined by the wavelength dependent measurement of the complex reflectance ratio

$$\rho =\frac{r_{p}}{r_{s}}=\tan\left(\Psi \right)e^{i\Delta }$$

where $r_{p}$ is the complex reflectivity of the p polarized light and $r_{s}$ is the complex reflectivity of the s polarized light. The result is that the amplitudinal component is related to Ψ and the phase information is captured in Δ. Figure 4 shows a simulation of a silicon wafer with a native oxide layer followed by mAb adsorption over a range of typical adsorbed amounts. One can see a subtle shift in the Ψ parameter and a larger shift in Δ.

A limitation of this technique is that *a priori* knowledge of the system is required as data analysis is indirect, therefore system modelling is required to extract dielectric properties. This can be done using software such as CompleteEASE (as was used for the data in Figure 4). A great strength of spectroscopic ellipsometry is that an accurate acquisition takes only seconds. This means that it is an ideal technique for dynamic *in-situ* measurements of the adsorbed amount of protein on reflective interfaces.

Neutron reflection is a versatile technique that can be used to characterise the thickness and hydration of protein films at air/water, solid/water and liquid/water interfaces. It is a non-destructive and powerful technique that can be used to probe thin layers on different interfaces. Neutrons, which are uncharged, can often penetrate matter because they only interact with the nucleus, which only occupies a tiny volume of atoms. As the neutron scattering power for atoms appears to vary rather randomly, neutrons can be used to distinguish neighbouring elements in the periodic table easily. Also, the scattering power of different isotopes can vary drastically. A particularly useful case is the different scattering power between H and D in amplitude and phase. As neutrons have a non-zero spin they have a response to external magnetic fields. This can be exploited for protein adsorption on magnetic metal surface such as stainless steel, adding additional information from parallel polarized neutron measurements. This improves model fitting and model constraints as there is a requirement that a single structural model fits both data sets.

In a typical neutron reflection experiment, a highly collimated beam of neutrons (polarized or non-polarized) is incident at a grazing angle θ and reflected at the same angle. The ratio of incoming and reflected beam intensities is termed neutron reflectivity (R) that changes with the incident angle and the wavelength of the neutron (i.e., how fast it is traveling). The momentum transfer (Q), a measure of change in momentum between reflected and incident neutrons, is defined as $Q=\frac{4\pi \sin\theta }{\lambda }$, showing that a range of Q can be measured by varying θ (incidence angle) and/or λ (wavelength). Pre-characterizing the interface in the absence of protein can then be used as a foundation to determine the structural details of the adsorbed protein. The thickness, volume fraction and roughness of the adsorbed protein layer can then be extracted from model fitting. It is therefore important to use a stable interface.

A major advantage from neutron reflection is the use of isotopic substitution of H by D due to their different scattering properties (D has a positive scattering amplitude or scattering length of 6.67 × 10^−5^ Å and H has a negative scattering length of −3.74 × 10^−5^ Å). Scattering length densities for C, O, N are 6.65 × 10^−5^ Å, 5.80 × 10^−5^ Å and 9.40 × 10^−5^ Å, respectively. Scattering lengths are additive, which means that the scattering length for H_2_O is −1.68 × 10^−5^ Å and that for D_2_O is 19.1 × 10^−5^ Å. Because a water molecule is about 30 Å^3^, the scattering length density (SLD) for H_2_O is −0.56 × 10^−6^ Å^−2^ and that for D_2_O is 6.35 × 10^−6^ Å^−2^. When 8.1% of D_2_O is mixed with H_2_O, the mixed water has zero scattering length density and is called null reflecting water (NRW). If neutron reflectivity is measured at the air/NRW interface, its value would be close to zero. On the other hand, if a surfactant or a protein adsorbs, the reflectivity arises from the adsorbed layer. Because there is no other component that contributes to the signal, reflectivity in this case can be converted to layer thickness and area per molecule directly when a uniform layer model is applied. Table 1 has the SLD of common materials used in studying protein adsorption using neutron reflection.

**Figure 5:**
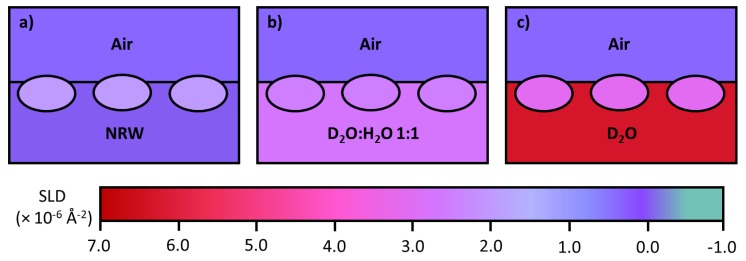
Schematic depictions of monoclonal antibodies (mAb) adsorption on surface of water: (**a**) null reflecting water (NRW) (determining the whole layer thickness), (**b**) H_2_O:D_2_O = 1:1 (close to the SLD of mAb, determining the thickness of the region above water) and (**c**) D_2_O (determining the extent of immersion in water).

**Figure 6:**
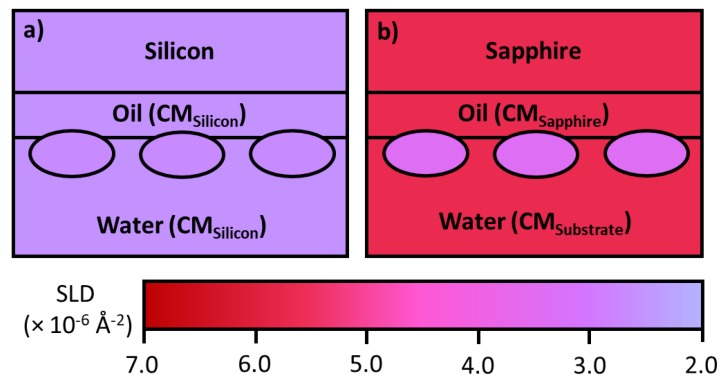
Schematic representation of mAb adsorption at the oil/water interface using (**a**) silicon as the substrate for the oil (excluding the silicon oxide layer for simplicity). There is very low contrast between the protein and other silicon matched components. (**b**) Sapphire is used as the substrate. The oil and other components are contrast matched to the substrate, this results in a much larger signal from the protein than from the silicon system.

Parallel measurements in D_2_O and a 1:1 mixture of D_2_O and H_2_O are commonly used in protein adsorption at the air/water interface as it provides information about the protein layer volume fraction and surface penetration. The isotopic contrasts created by varying D_2_O and H_2_O are schematically shown in Figure 5. Another practical use of isotopic contrasts in neutron reflection is the ability to measure mAb adsorption at the oil/water interface. Often a silicon block is used as a substrate but sapphire is preferred in this case due to its greater SLD (5.75 × 10^−6^ Å^−2^) enabling a better contrast between it and the protein, schematically depicted in Figure 6.

**Figure 7:**
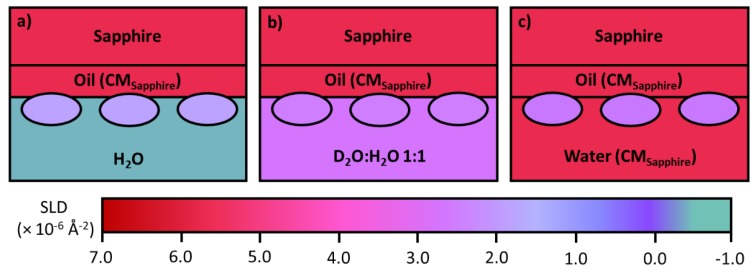
Schematic diagram of protein adsorbed to the oil/water interface using a sapphire substrate. (**a**) H_2_O water phase for information about the entire interface. (**b**) D_2_O:H_2_O 1:1 highlights mixing between the protein and the oil. (**c**) All the systems components (except the protein) contrast matched to sapphire to extract the adsorbed amount of protein at the interface.

Stable hexadecane oil films can be formed on hydrophobic sapphire substrates, via spin coating. A layer with a thickness around 1 µm can be produced to mimic bulk oil. For NR there is only a modest loss of neutron beam intensity as a result of attenuation from the oil layer due to its finite thickness. After the film is formed, it is frozen before it is assembled with the liquid trough and filled with buffer or solution under appropriate contrasts. To avoid the complication from reflection at the solid/oil interface, the SLD of the oil is also brought to 5.75 × 10^−6^ Å^−2^, the same as that of the sapphire. If the SLD of the buffer solution is kept the same as well, the only signal will be from the mAb layer adsorbed at the oil/water interface, as seen in Figure 7c. As the solvent is invisible, this contrast doesn’t contain information about the mixing of the mAb with oil or water, only the amount of adsorption. A parallel run in the buffer solution contrast matched to the mAb (SLD = 2.56 × 10^−6^ Å^−2^, close to that from 1:1 ratio of H_2_O and D_2_O) enables us to determine the extent of mixing of the mAb into the oil, Figure 7b. Finally, a further measurement in H_2_O contains all structural information about the interface, shown in Figure 7a. The combined resolution of neutron reflection with all three contrasts leads to the information about the mixing of the mAb in the aqueous side of the interface, resulting in a comprehensive description of adsorption at the oil/water interface.

#### 3.2.2. Imaging Techniques

SEM and AFM are scanning probe microscopic techniques, which can obtain images of surfaces by scanning the surface with a probe. The probe of SEM is a focused beam of electrons. The intensity of the interaction between the electrons and a given surface can be collected to produce an image. However, special sample preparation is needed for most biomaterials to increase their electrical conductivity. AFM is more straightforward and applicable in the study of mAb adsorption, which gathers the surface information by “touching” it with a mechanical probe [41]. AFM can be used to measure the thickness of adsorbed protein films as well as some resolution in the X-Y plane.

In addition to high-resolution imaging, AFM also can provide mechanical measurements via different choices of tips and modes of contact, e.g., tapping mode or non-contact mode. A non-contact mode is often selected to avoid damaging protein molecules, but the resolution of the images to be obtained is limited. In general, a sharp tip is used for imaging and a flattened tip for force measurement. The tip is mounted on a cantilever and is brought close to the surface of interest. The interactive force between the tip and the surface deflects the cantilever. From the deflection, an image of the surface can be built up as the tip scans across the surface [41].

In tapping mode, the cantilever is driven to oscillate at its resonant frequency with variations between the driving oscillation and the resulting oscillation, indicating changes in the surface properties, i.e., changes in height and adhesion. When the oscillating tip is brought close to the surface, the forces acting on the tip (van der Waals force, electrostatic interaction and so on) attenuate it from the constant amplitude. Vilhena et al. have used AFM in liquid and molecular dynamic simulations to investigate antibody adsorption to graphene. They found that some of the IgG antibody molecules adsorbed vertically, offering an increased number of Fab domains to solution [42].

Quartz crystal microbalance with dissipation (QCM-D) has been used to study the reversibility of the primary layer of protein adsorption as well as the more diffuse secondary protein layer of therapeutic antibodies on stainless steel [43]. Others have used it to investigate the influence polysorbate 80 (PS80) has on the adsorption of two different mAbs [44]. It was observed that the antibodies readily adsorbed to the hydrophobic surfaces but PS80 could help prevent adsorption if pre-adsorbed. This study aimed to investigate conditions similar to those in infusion bags. QCM-D works by exploiting the piezoelectric nature of quartz. A sensor made out of quartz is forced to oscillate by applying an AC electric field. When the field is removed the resulting decay frequency can be used to infer the mass on the sensor (including hydrodynamically coupled water) and the change in dissipation can be used to infer the viscoelastic properties of the bound material [45]. This is ideal for studying the absorption/desorption dynamics of protein as well as its interaction with other excipients.

### 3.3. Studies of Adsorption of Natural Model Proteins

Many studies have examined adsorption of naturally occurring proteins at the air/water interface [46,47] and the silicon oxide(SiO_2_)/water interface [48,49,50]. These studies investigate how adsorbed protein layers at model interfaces evolve with time and with varying mAb solution concentration using spectroscopic ellipsometry and neutron reflection. The impact of protein surface charges is widely demonstrated by changes of solution pH with respect to the pI of the protein. Changes in the hydrophobicity of the substrate surface have been examined using self-assembled monolayer (SAM) anchored onto silica via silane chemistry. Proteins such as lysozyme and albumins tend to adsorb similar amount at the bare SiO_2_/water and OTS (octadecyltrimethoxysilane) modified SiO_2_/water interfaces, but the hydrophobic surface tends to cause structural unfolding [51]. In contrast, a SAM bearing terminal hydroxyl groups affords intermediate hydrophobicity, but such surfaces can substantially reduce protein adsorption [52]. Surface coatings bearing phosphorylcholine (PC) groups in the form of a SAM or grafted polymeric film can also reduce protein adsorption substantially due to the strong hydration of the PC [53,54,55,56]. These studies are highly informative with regards to the reduction of surface and interfacial adsorption of proteins and control of their structural stability.

A number of studies have also examined the co-adsorption of surfactant and protein, as these studies can inform us about possible competitive processes and structural impact to proteins [56]. Nonionic surfactants such as C_12_E_5_ (pentaethylene glycol monododecyl ether) can prevent lysozyme adsorption at the air/water interface because of its greater surface activity [57]. Generally, nonionic surfactants do not cause structural deformation of protein. However, the co-adsorption of lysozyme with ionic surfactants such as sodium dodecylsulphate (SDS) is far more complex because protein-surfactant interactions can be driven by favourable electrostatic interactions [58]. With increasing surfactant concentration, the number of ionic surfactant molecules associated with each lysozyme increases which facilitates unfolding. As the net charge on lysozyme molecules decreases, precipitation occurs. Bound molecules become saturated with SDS and the concentration of monomer surfactant molecules starts to rise at the surface overtaking and ultimately replacing the surface active surfactant-protein complexes. At the SiO_2_/water interface, SDS binding can help remove pre-adsorbed lysozyme [59], but also unfolds protein. In contrast, nonionic C_12_E_5_ does not co-adsorb at the solid/water interface and did not remove pre-adsorbed lysozyme or albumins [60]. These studies thus demonstrate very different effects of nonionic and ionic surfactants to the structural stability of proteins and competitive adsorption.

### 3.4. Adsorption of IgG Proteins

Adsorption characteristics of humanized IgG1 or its close homologues with small sequence modifications have been extensively investigated by both spectroscopic techniques and imaging techniques. Xu et al. have examined how a pair of variable domain modified mAbs adsorb at the SiO_2_/water interface [61,62,63]. From AFM imaging, they observed some dimerization of the adsorbed mAb molecules. Neutron reflection revealed the flat-on adsorption with the thickness of the adsorbed layers around 40 Å. Increased concentration led to increased interfacial adsorption as the volume fraction of the adsorbed protein increased. Although the total adsorbed amount increased the total thickness only increased slightly (to 45–50 Å). This implies that the orientation of adsorption was not impacted drastically, only how the protein packed at the surface. Further work by Zhao et al. on the adsorption of prostate antigen binding mAbs showed rather similar adsorption behaviour [64,65]. These studies demonstrate similar adsorption dynamics, adsorbed amount, adsorbed layer thickness and secondary structure changes over a range of proteins at interfaces such as air/water, glass/water, and oil/water interfaces.

Using neutron reflection, structural features of adsorbed protein molecules, including deformation and hydration within the protein layer can be determined through accurate measurements of layer thickness and composition [12,38,39,64]. Neutron reflection is particularly sensitive to inhomogeneity perpendicular to the surface. Therefore, it can be an effective tool for detecting unfolding of the adsorbed molecules. Other techniques such as surface Fourier transform infrared spectroscopy have been used to determine secondary structure changes in adsorbed protein molecules. Most studies tend to implement several techniques to acquire different properties of a given protein.

## 4. Recent Advances in Adsorption Studies of Bioengineered mAbs at Different Interfaces

With mAbs now established as important therapeutic drugs there is an imperative to understand how to control their adsorption during manufacture to ensure a stable drug product [66]. As discussed, it is hypothesized that mAbs, exposed to siliconized glass or plastic surfaces of a primary container, may adsorb/desorb and so aggregate through structural change. There is a lack of understanding of the adsorption, desorption, layer formation and induced aggregation at interfaces and subsequently the means to mediate them and control their adverse influences.

### 4.1. The Air/Water Interface

**Figure 8:**
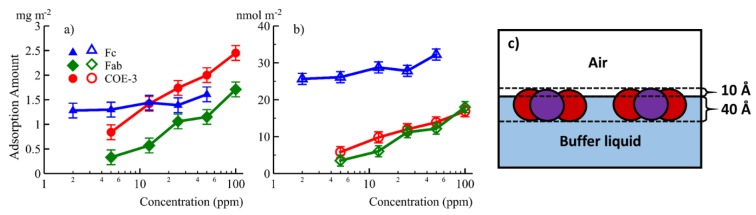
The equilibrated amount of adsorption in mg/m^2^ (**a**) and nmol/m^2^ (**b**) plotted against concentration for Fc (▲), Fab (♦) and the whole mAb COE-3 (●). (**c**) A schematic representations of the surface adsorbed COE-3 layers, Fab represented in red and Fc in purple. Reproduced with permission from Z. Li, ACS Applied Materials & Interfaces, 2017.

An interface commonly encountered in biopharmaceutical production and storage, so it is important to study interfacial adsorption and desorption of mAbs. Unfortunately, it is difficult to gain basic information such as the changes of adsorbed amount and layer thickness with time. We have studied the adsorption of COE-3 at the air/liquid interface using neutron reflection and surface tension experiments under different buffer conditions (mAb concentrations, pH and ionic strengths) [67]. We have also isolated and investigated the adsorption of the Fc and Fab segments of COE-3 [35]. The results implied that Fc and Fab have different interfacial properties, as shown in Figure 8a,b. Fab dominated the concentration-dependent mAb adsorption while both Fab and Fc contributed to the amount of mAb adsorbed. Using multiple contrasts (NRW, Contrast-Match-Antibody water (CM2.58) and D_2_O), the adsorbed layer structure and its immersion depth was revealed, as shown in Figure 8c. Our neutron reflection study has shown that NRW and CM2.58 could offer significant benefits in emphasising mAb and mAb fragments (e.g., Fab and Fc) at the interface by showing the entire adsorbed layer and the region out of the water surface, respectively. The Fc layer remained constant at 40 Å while Fab and mAb layers increased very little from 45 to 50 Å when mAb concentration increased from 5 to 50 ppm. On the other hand, the volume fraction of the adsorbed antibody increased from 40% to 56%, this results in quite a drastic change in adsorbed amount, as can be seen in Figure 8. These studies implied that the adsorbed mAb, Fc, and Fab all retained their globular structures and were oriented with their short axial lengths perpendicular to the air/liquid interface. The same measurement in D_2_O revealed how the adsorbed protein layers mixed with water. The results showed that for COE-3, its Fab and Fc were predominantly immersed in water once adsorbed.

The adsorption and desorption of a number of mAbs with or without nonionic surfactants at the air/liquid interface has been studied using a variety of methods. For example, Shieh and Patel [68] measured the surface pressure changes upon surface adsorption for 16 IgG mAbs. They showed that their approach predicted the agitation-induced mAb aggregation. Adsorption of the mAbs to the air/water interface can enhance agitation induced aggregation, with the initial rate of surface pressure rise correlating with aggregation. Interestingly, the authors showed that other factors such as mAb hydrophobicity, equilibrium surface pressure, and its adsorbed amount did not correlate with aggregation. They further showed that mAb rearrangement and conformational change upon surface adsorption drove attractive mAb–mAb interactions. Their work demonstrated the relevance of kinetic surface pressures to predict the propensity of aggregation associated with surface adsorption. Dilatational rheometer with simultaneous pressure and bubble coalescence can help examine the stability of mAbs under interfacial ageing. Using mAb 1 (a fully humanized IgG1 from AstraZeneca, Gaithersburg, MD, pH = 6.0 and pI = 8.15), Lin et al. [69] showed that dilatational surface deformations are more important to particle formation than shearing under the constant area through both bulk shearing and interfacial shearing (studied via fluorescent dye detection of subvisible particle concentration). Further work from the same group [70] examined the stability of 2 mAbs mixed with polyethylene glycol (PEG). The mAb with greater aggregating tendency coadsorbed with PEG at the interface while the other one was replaced by PEG from the interface. Imaging techniques like fluorescence microscopy and AFM have been recently developed to visualise the adsorbed antibody layer. An example for fluorescence microscopy was shown by Leiske et al. [71]. They labelled 2 mAbs with different surface activities using Nile red. The Nile red fluorescence for the mAb with higher surface activity increased immediately, whereas the fluorescence signal for the one with lower surface activity evolved much slowly. This confirmed that the suface activity plays an important role in the dynamic adsorption process. An AFM method demonstrated by Koepf et al. [72] transferred the surface adsorbed mAb films onto mica, with the surface films characterized being under adsorption, compression or decompression via Langmuir-Schaefer deposition, followed by underwater AFM scanning. The images revealed that compression led to the formation of wrinkles across the scanned areas and decompression resulted in even smoother films than the adsorbed mAb films.

**Figure 9:**
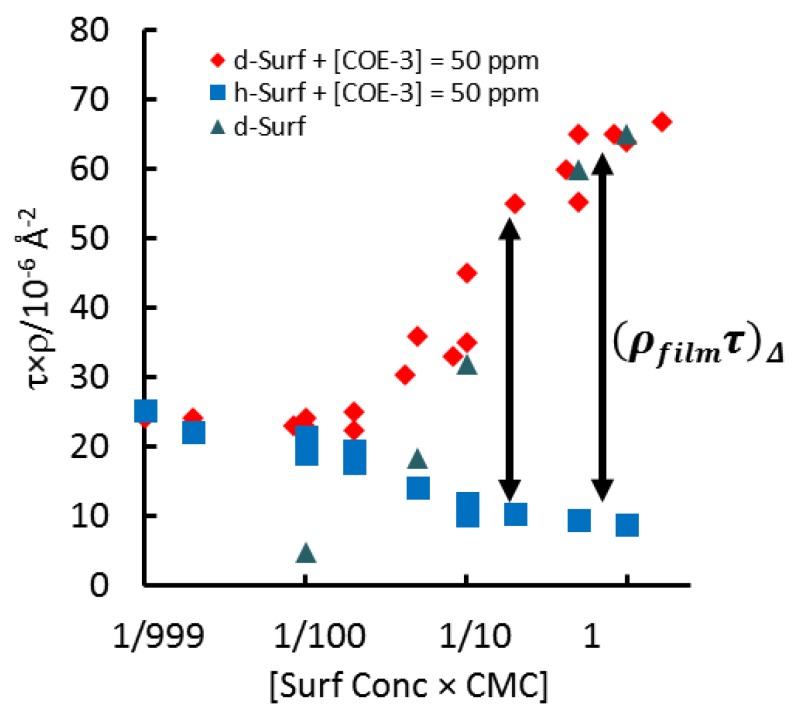
Plots of thickness × SLD (τρ/10^−6^ Å^−2^) versus the concentration of surfactant (expressed as the fraction of CMC) for both h-Surf and d-Surf, with the concentration of COE-3 fixed at 50 ppm. The product from the binary mixture of d-Surf and COE-3 is marked in blue diamonds ((τρ)_d-Surf_) and that from the mixture of h-Surf and COE-3 in red squares ((τρ)_h-Surf_). The τρ data from d-Surf alone are shown in green triangles. Reproduced with permission from Z. Li, mAbs; published by Taylor & Francis, 2017.

An attractive approach to prevent mAb adsorption on the surface of water is to add nonionic surfactant into the mAb solution. Nonionic surfactants do not tend to bind mAb to cause structural unfolding, but they are more surface active than mAbs and are expected to stop mAb adsorption by the competitive action. In our recent work [67], we mixed COE-3 with the nonionic surfactant, polysorbate 80 (PS 80) in different ratios, as it is the most common nonionic surfactant used in biopharmaceutical production. We undertook the neutron reflection measurements by fixing the mAb concentration at 0.050 mg/mL while varying the surfactant concentration from well below its CMC (critical micellar concentration) to 10 times the CMC, in both its hydrogenated and deuterated forms, in NRW. Measurements carried out under isotropic contrasts indicated that mAbs retained globular structure when either adsorbed alone or co-adsorbed with surfactants. But as the bulk concentration of the surfactant increases, its amount co-adsorbed on the surface increases. Figure 9 shows the plots of the product of τρ, where τ denotes the layer thickness and ρ denotes the total SLD of the layer, versus surfactant concentration. At the surfactant concentrations below 1/50CMC, there is little surfactant co-adsorption and the signals are consistent with the adsorption of the mAb.

However, as the surfactant concentration exceeds 1/50 CMC, the product of τρ starts to diverge, with the hydrogenated surfactant producing a reduced signal and the deuterated surfactant producing fast rising signal. These changes are consistent with the fast increase in the surfactant adsorption and their eventual replacement of the adsorbed mAb molecules. For reference, the adsorption from deuterated surfactant alone is also shown, the difference in signal from 1/5 CMC to 1/20 CMC indicates co-adsorption. This work shows that above 1/20 CMC, nonionic PS 80 suppresses surface adsorption of this mAb.

**Figure 10:**
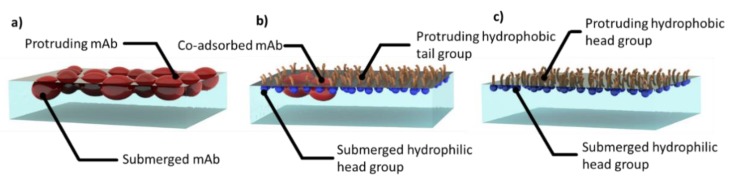
Schematic depiction of COE-3 adsorption at 0.050 mg/mL (**a**) on its own with a thickness close to 50 Å and most part of the layer being immersed in water, (**b**) co-adsorption with nonionic PS 80 surfactant (blue heads) when its concentration is below 1/20 CMC. (**c**) As the surfactant concentration moves above 1/20 CMC, the mAb is completely expelled by the surfactant.

Thus, the interfacial events of the competitive adsorption processes between mAb and the non-ionic PS 80 surfactant can be schematically illustrated in Figure 10. At the start of the experiment, the adsorption of mAb at its fixed concentration of 0.05 mg/mL forms an almost uniform layer of some 50 Å thick (Figure 10a). Upon addition of surfactant to 1/20 CMC, the surfactant co-adsorbs with mAb (Figure 10b), but as the surfactant concentration goes above 1/20 CMC, surfactant adsorption becomes dominant and then completely expels mAb from the surface (Figure 10c).

### 4.2. The SiO_2_/Water Interface

Native SiO_2_ formed on silicon makes for a good model to understand protein behaviour at glass/water interfaces due to the similarity to a glass surface. Because many natural proteins adsorb onto silica, it can be expected that bioengineered mAbs will adsorb as well but the difficulty lies in predicting the exact adsorbed amount, layer structure and conformational orientation etc. With many intertwined parameters current studies can only be limited to quite narrow measurement ranges with a limited prediction power.

Type I borosilicate glass vials and pre-filled syringes are widely used in the storage of protein biopharmaceuticals. It is thus useful to understand how bioengineered proteins like mAbs adsorb at the glass/water interface to understand how adsorption can impact protein stability. Silica has been widely used as a model for glass to facilitate many protein adsorption studies by different techniques. Techniques such as spectroscopic ellipsometry and neutron reflection benefit greatly from an incredibly flat and reflective surface [2,48]. It is common to deploy several different techniques in a given study to try to obtain complementary information. While spectroscopic ellipsometry can measure how the adsorbed amount changes with respect to bulk mAb concentration, pH, ionic strength and time, AFM reveals the structure of adsorbed molecules and interfacial Fourier transform infrared spectroscopy reveals the secondary structure changes at the same interface.

One area of interest is to design coatings that can manipulate protein adsorption. Couston et al. investigated the adsorption of a mAb onto a silanized silica with octadecyltrichlorosilane (OTS) to generate octadecyl monolayer [73]. This is a mimic of a hydrophobic plastic surface and they showed that adsorption was greatly reduced at the hydrophobic surface with only 2 mg/m^2^ of mAb adsorbed, compared to around 5.5 mg/m^2^ on the bare SiO_2_ surface It was also shown that standard PS 80 could displace roughly 50% of the adsorbed protein on the hydrophobic surface due to an interaction between the fatty acid tail of the polysorbate and the modified surface. Benefits from coatings need to be compared with the risks of introducing potential leachables which if not properly handled can be a safety concern [74].

Pan et al. have studied dynamic adsorption of COE-3 at the SiO_2_/water interface using spectroscopic ellipsometry and neutron reflection. They compared their experimental data with simulations based on the DLVO theory [75]. The dynamic adsorption of this mAb was characterized by an initial rapid increase followed by a plateau. They found that the initial rates of the dynamic adsorption were strongly correlated to the bulk concentrations in the range of 0.002–0.2 mg/mL, with the adsorbed amount reaching the plateau about 2.2 mg/m^2^ (pH 5.5). They showed that the simulated results replicated the equilibrated COE-3 adsorption very accurately: this is the steady state where desorption and adsorption fluxes of the mAb molecules within the adsorbed plane are equal. Increase in pH from 5.5 to 9 led to an increase in the adsorbed amount to about 3 mg/m^2^ at 0.02 mg/mL, showing the strong impact of lateral repulsion in the interface. On the other hand, increase in ionic strength led to reduced adsorption, and simulations indicated the electrostatic screening by ions, apart from mAb/SiO_2_ attractive binding and lateral repulsion. While neutron reflection and ellipsometry data were well consistent in adsorbed amount, neutron studies revealed that the adsorbed layers were close to 50 Å thick even though the adsorbed amount varied substantially. This rather constant range of thicknesses indicated that Fab and Fc fragments are in direct contact with the substrate, forming a uniform layer with minimal structural disruption. At pH 9, the adsorbed amount could reach 3.6 mg/m^2^ at 0.02 mg/mL COE-3, with a high volume fraction of 0.5.

**Figure 11:**
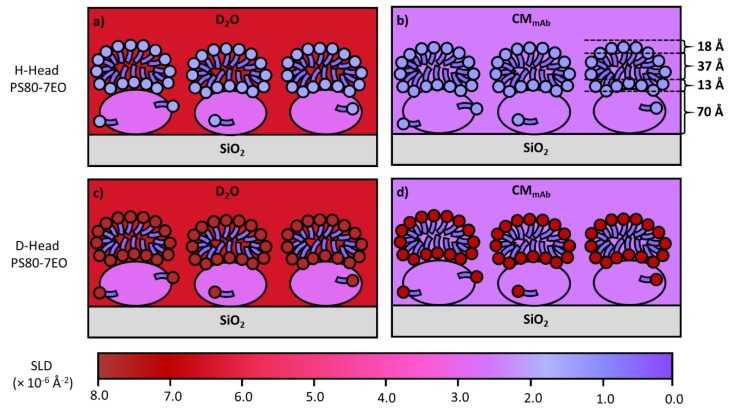
A schematic representation of the use of isotopic contrasts to highlight the structure of the interfacial layers formed by self-assembly of PS80-7EO on top of a COE-3 layer at the SiO_2_/water interface. Schematic (**a**) depicts the system in D_2_O solvent coupled with hydrogenated (H-PS80-7EO) nonionic surfactants, (**b**) depicts the system in CMAb (contrast matched to COE-3 with SLD = 2.56 × 10^−6^ Å^−2^) solvent coupled with hydrogenated (H-PS80-7EO) nonionic surfactants, (**c**) depicts the system in D_2_O solvent coupled with head deuterated (D-Head PS80-7EO) nonionic surfactant and (**d**) depicts the system in CMAb (contrast matched to COE-3 with SLD = 2.56 × 10^−6^ Å^−2^) solvent coupled with head deuterated (D-Head PS80-7EO) nonionic surfactants. The model schematics were based on simultaneous fittings of 4 reflectivity profiles measured under the 4 isotopic contrasts at the SiO_2_/water interface. COE-3 adsorption at 0.010 mg/mL mixed with 0.20 mg/mL protonated or deuterated PS80-7EO.

Nonionic surfactants such as PS 80 are routinely added into mAb solutions during formulation to mitigate against particulate formation during shake/stir stresses. But it is worth noting that polysorbates can degrade and depending on the degradation root (hydrolysed polysorbate) can result in an increase in particle formation during shaking stress [76]. A study by Kim et al. [77] showed that polysorbates were able to inhibit protein adsorption but did not result in a protein/surfactant complex with an overall lower surface activity. To add to this, Li et al. [78] have recently shown that PS 80 and C_12_E_5_ could not prevent COE-3 adsorption at the SiO_2_/water interface. When a polysorbate 80 analogue with 7 ethoxylates (PS80-7EO) was used, it co-adsorbed but did not affect the equilibrated amount of adsorption from COE-3. Spectroscopic ellipsometry was very useful when following the dynamic processes by measuring the changes in the total adsorbed amount via either co-adsorption (both surfactant and COE-3 were added to the system simultaneously) or sequential adsorption. The different dynamic changes could be well monitored within a time resolution of a minute by this approach. Through the event mode, neutron reflection could also monitor the time-dependent changes within a time resolution of 3–4 min for acceptable statistical quality. The surfactant showed little adsorption at the initial stage. In contrast, COE-3 adsorption was influenced only slightly by the presence of the surfactant. At the late stage of dynamic co-adsorption, COE-3 experienced structural rearrangement with PS80-7EO, resulting in the COE-3 layer still bound on the SiO_2_ surface but with a well-defined surfactant bilayer self-assembled on the top. The interfacial layer eventually consisted of an inner COE-3 layer of 70 Å thick and an outer surfactant bilayer of a further 70 Å. There were clear boundaries at the mAb-surfactant and surfactant-bulk water interfaces. Figure 11 shows the schematic representations of the final interfacial layers formed via co-adsorption. The structural parameters were obtained through simultaneous fittings to the 4 reflectivity profiles measured under 4 different isotopic contrasts involving the 2 solvent contrasts of D_2_O and CMAb (water contrast-matched to COE-3 with SLD = 2.56 × 10^−6^ Å^−2^) and hydrogenated and deuterated PS80-7EO surfactants. The self-assembled interfacial layers were found to be robust and once formed, they could prevent further mAb adsorption, desorption and further structural rearrangement. It was anticipated for such robust interfacial layers to exist for formulated mAbs stored in soda lime glass, but it would be useful to further explore the behaviour of similar layers formed in siliconized glass syringes.

Mazzer et al. [79] investigated IgG4κ adsorption at the silica/water interface. The adsorption resulted in a side-on orientation at pH 4.1 and a 60 Å thickness. Using 2 isotropic solvent contrasts (D_2_O and H_2_O) enabled a higher level of confidence in the fit. Further work investigated the protein-antibody complex used to mimic an affinity chromatography surface with results suggesting that two IgG molecules would bind in a skewed orientation to the protein at the silica/water interface. This highlights the power of neutron reflection to investigate the orientation of antibody adsorption in complex systems.

### 4.3. The Stainless Steel/Water Interface

Interest in this interface stems from its practical relevance. It has been suggested that stainless steel can induce structural damage to adsorbed mAbs leading to aggregates in the bulk protein solution. Structural changes have been observed from the work of lysozyme or bovine serum albumin (BSA) adsorbed to different types of stainless steel as well as therapeutic fusion proteins [43,80]. These studies have also shown that adsorbed proteins could compromise the corrosion resistance of the steel surface and enhance metal ion release (up to 40-fold increase for BSA). These phenomena could potentially be alleviated if there were either a way to prevent protein adsorption or even make it occur in a more controlled manner. Furthermore, stainless steel has been observed to mediate protein aggregation. Stainless steel piston pumps we seen to produce particle formation on a novel fusion protein whereas this was not the case when a ceramic piston pump was involved [43].

Using the NISTmAb (an IgG1 mAb reference material from the National Institute of Standard and Technology), Kalonia et al. [81] revealed that the amount of subvisible particles (SVP) induced by stainless steel was substantially more than from alumina. SVPs tended to form more easily under extreme shear stresses. These authors also found that as mAb concentration increased SVPs were easier to produce. Intriguingly, only a small fraction of desorbed mAbs shed into solution could induce substantial SVP production. Neutron reflection helped reveal a high amount of the mAb deposited at the stainless steel interface over a wide concentration range. Shear stress could remove adsorbed mAbs leading to the formation of subvisible particles. The NR and quartz crystal microbalance studies also revealed that over a short time scale low and high mAb concentrations led to similar adsorption. Over a long time period the adsorption at high concentrations continued whilst adsorption at low concentrations plateaued. Other studies have highlighted similar issues with material incompatibility [82]. Bee et al. suggest that storage of biological products over a two year period would result in a small loss in protein mass but could still result in subvisible particles that could compromise the safety of the biotherapeutic if not handled correctly.

Modifying steel surfaces could be one way of tackling the unwanted phenomenon associated with protein adsorption to stainless steel surface. It has been shown that coating a polycaprolactone film onto 316L stainless steel can effectively reduce the amount of adsorbed BSA as determined via a BCA protein assay [83]. Surface modifications could provide an effective way of limiting adsorption and potentially alleviate some undesirable repercussions of protein adsorption onto stainless steel. As with any coating the risk of introducing leachables become a concerning issue [74].

### 4.4. The Oil/Water Interface

Relevant to the use of silicone oil as a lubricating film for pre-filled syringes as outlined previously. Neutron reflection has been used to study protein adsorption at the oil/liquid interface [84]. The technique involves a substrate such as quartz or sapphire to be used as a vehicle to create a stable oil/water interface. To reduce attenuation of the neutron beam the oil should be a thin layer, typically around 1–2 µm, but the challenge lies in keeping the structure of the film stable during the entire experiment. As explained previously, oil contrast matched to the solid substrate must be used to avoid a large signal from the oil/solid interface. Polydimethylsilance (PDMS) oil has been widely developed for lubricating the mAb pre-filled syringes, but hexadecane is used as a model oil at this stage to help develop the experimental capability, as it is more stable at the interface. Changes in water contrasts help highlight the adsorbed mAb layer differently, resulting in the information about the adsorbed amount, mAb layer thickness, its extent of mixing with oil and water. Zarbakhsh et al. [84] demonstrated the ability to resolve interfacial structures at the oil/water interface using neutron reflectometry with further work by Campana et al. [85] investigating the adsorption of BSA at the water/hexadecane interface. Results for BSA adsorption showed that the protein formed a relatively thick (75 Å) layer in the oil phase, suggesting a hydrophobic region of the protein freely to interact with the oil resulting in changes in secondary and tertiary structures [85]. Recently, Ruane et al. [86] investigated the adsorption of COE-3 at the water/hexadecane interface using almost the same setup as developed by Zarbakhsh et al. As schematically illustrated in Figure 6, the SLD of the oil was contrast matched to that of the sapphire, thus making the sapphire/oil interface invisible to neutrons. The thickness of the oil film was fixed at about 1 µm, to balance between having a sufficiently thick oil film and modest attenuation, with the entire adsorbed amount of COE-3 quantified by the contrast matched oil run (Figure 7c), the extent of mixing of the protein into oil by contrast matched to COE-3 (Figure 7b) and any extent of mixing into water by the H_2_O run (Figure 7a). The results show very little to no penetration of the mAb into the oil interface. Although a uniform protein layer modelled the main trend of the reflectivity data, it was non-ideal. Modeling the adsorbed mAb as two-layers led to the proposition of the dominant flat-on orientation of 45 Å thick with some minor ones being tilted forming a more sparse protruding layer of 20 Å thick. This work paves the way for future studies using PDMS, a substance used to lubricate syringe plungers.

## 5. Conclusions and Future Prospects

With the increasing number of therapeutic mAbs, bispecific Abs, antibody drug conjugates, and fusion proteins, there is an ever increasing need to assess and predict stability by different means. These studies will feed information back to help alleviate various technical challenges in mAb fill/finish, shipping and storage, and other applications such as biosensors. This review has focused on the introduction of surface and interfacial adsorption of mAbs at industrially relevant interfaces. The use of different model interfaces allows a systematic examination of the adsorption behaviour from a given mAb under well-defined solution conditions. The selection of different methods enables us to gain complementary structural information. In the cases of solid/water and oil/water interfaces, the selection of different substrate surfaces allows us to mimic the chemical nature of the material that is used in commercially relevant applications.

While various imaging techniques provide morphological details of substrate surfaces before and after mAb adsorption, spectroscopic techniques such as neutron reflection have shown a strong competitive edge for in situ characterisation. Neutron reflection will continue to contribute to this area of development through better deployment of deuterated or partially deuterated material including polymeric substrates and mAbs, thereby enhancing the sensitivity and resolution of neutron reflection. For example, while the role of PDMS oil in lubricating the barrels of pre-loaded syringes is well established, there may well be other consequences such as partial unfolding of adsorbed mAbs. Although the impact of the PDMS oil on the structural integrity of adsorbed mAbs can be studied by neutron reflection, deuterated PDMS should also be used to form a deuterated PDMS oil film with SLD matching the solid substrate such as quartz or silicon to avoid reflection from the solid/oil interface. In addition, expressing deuterated mAbs and mAb fragments could open up new opportunities for neutron reflection measurements under different isotopic contrasts. These additional runs will enable us to obtain more precise information about how exactly mAb molecules adopt their conformations once adsorbed, thereby enhancing structural resolution.

Although exact structural features must still be obtained from fitting neutron reflection data, the neutron data with enhanced resolution measured under parallel isotopic contrasts could challenge in situ AFM imaging studies. It must be however emphasised that neutron reflection is not a bench-top technique. Because neutron instruments are centralized, all neutron experiments must be carried out at large user facilities such as ISIS at Rutherford-Appleton Laboratory, UK, Institut Laue-Langevin at Grenoble, France and NIST Center for Neutron Research at Gaithersburg, USA, through competitive grant applications and their access is limited for the time being. Facilities such as these are continuing to increase neutron beam fluxes and developing improved experimental conditions, thus increasing their efficiency can accommodate more experiments within limited access times available. Prior studies from AFM, spectroscopic ellipsometry and interfacial FTIR measurements can make neutron reflection work better defined and the results together complement each other.

An evolving obstacle in the course of complementary studies lies in the integration of the data from different techniques. Computer modelling will play a pivotal role in harmonizing these data structurally and dynamically providing extra supporting information about conformation of adsorption. Molecular dynamics and Monte Carlo simulations have gained a huge momentum over the past few years in studying protein adsorption [38,39,40,87]. Although the use of computer modelling in studying mAb interfacial processes is currently limited, it is envisaged that once computer modelling becomes better integrated with experimental studies, it will help improve protein adsorption predictions and guide experimental plans. Their combined use over the next 5–10 years will substantially improve our knowledge about mAb adsorption and desorption. This new level of understanding will directly benefit mAb design and stabilisation.

## Figures and Tables

**Table 1:** Useful neutron scattering length densities of common substances used in neutron reflectivity. The scattering length density (SLD) of COE-3 changes (as with all proteins) depending on the ratio of D_2_O/H_2_O in solution. Labile hydrogen/deuterium exchange results in higher SLD when a larger proportion of solution is D_2_O.

Material	D_2_O	H_2_O	Silicon	SiO_2_	Sapphire	Quartz	NRW	COE-3 (CM_COE-3_)
SLD (Å^−2^ × 10^6^)	6.35	−0.56	2.07	3.47	5.75	4.17	0.00	2.56
